# Knowledge gaps in psychedelic medicalisation: Clinical studies and regulatory aspects

**DOI:** 10.1016/j.nsa.2024.103938

**Published:** 2024-01-11

**Authors:** Drummond E-Wen McCulloch, Matthias E. Liechti, Kim PC. Kuypers, David Nutt, Johan Lundberg, Dea Siggaard Stenbæk, Guy M. Goodwin, Gerhard Gründer, Florence Butlen-Ducuing, Marion Haberkamp, Steffen Thirstrup, Gitte M. Knudsen

**Affiliations:** a Neurobiology Research Unit, Rigshospitalet, University of Copenhagen, Denmark; b Division of Clinical Pharmacology, University Hospital Basel, Basel, Switzerland; c Department of Neuropsychology and Psychopharmacology, Faculty of Psychology and Neuroscience, Maastricht University, Maastricht, the Netherlands; d Centre for Psychedelic Research, Department of Brain Sciences, Imperial College London, London, UK; e Centre for Psychiatry Research, Department of Clinical Neuroscience, Karolinska Institutet, & Stockholm Health Care Services, Region Stockholm, Karolinska University Hospital, SE-171 76, Stockholm, Sweden; f Department of Psychology, University of Copenhagen, Copenhagen, Denmark; g University Department of Psychiatry, Oxford and Compass Pathways Plc, London, UK; h Central Institute of Mental Health, Department of Molecular Neuroimaging, Medical Faculty Mannheim, Heidelberg University, Mannheim, Germany; i European Medicines Agency, Amsterdam, the Netherlands; j Federal Institute of Drugs and Medical Devices, Section Neurology, Psychiatry, Ophthalmology, Bonn, Germany; k European Medicines Agency's Central Nervous System Working Party (CNSWP), the Netherlands; l Institute of Clinical Medicine, University of Copenhagen, Copenhagen, Denmark

## Abstract

Psychedelic drugs including psilocybin and LSD are undergoing clinical trials for a range of psychiatric and neurological conditions, and have particularly shown substantial promise in phase 2 studies of depression. In this article we outline key knowledge gaps that may be imperative for a successful implementation of psychedelic drugs as medicines as identified by members of the European College of Neuropsychopharmacology at the New Frontiers Meeting in Nice (2023). Key themes include pharmacokinetic and pharmacodynamic characterisation, comparisons between psychedelics, the relation between the duration of subjective effects and therapeutic outcomes, polypharmacology, and the impact of psychological support. We conclude with a perspective from the European Medicines Agency and Heath Technology Assessors on the most pressing requirements for medical implementation in Europe.

## 1 Introduction

In part one of this perspective concerning the knowledge gaps in the development of psychedelics as medicines identified by the European College of Neuropsychopharmacology's New Frontiers meeting in Nice March 2023, we consider preclinical and clinical neuroimaging research to unravel the therapeutic mechanism of action of classical psychedelic drugs which are serotonin 2A receptor (5-HT2AR) agonists that produce dose-dependent LSD-like subjective effects in humans, though they often have affinity for several other receptors.

As highlighted in part one, classical psychedelics have shown substantial early promise in a range of psychiatric conditions and psilocybin is now moving into phase 3 for the treatment of treatment-resistant depression, with several related compounds and indications set to follow in the coming years. There is a clear need for larger cohort samples and more rigorously designed clinical trials to show the safety, efficacy, and cost-effectiveness of psychedelic drugs as medicines. Yet, at this early stage, clinical work can be performed to better understand the nature of clinical effects from a pharmacokinetic, pharmacodynamic, and precision medicine perspective. By deepening these key basic understandings, we can help to inform future large clinical trials, maximising their chance of success.

## 2 Phase 1 clinical trials

### 2.1 Inter-individual differences

Phase 1 studies in healthy subjects are essential to define the pharmacokinetics (PK), pharmacodynamics (PD), pharmacogenetics, metabolism, elimination, and tolerability of novel and classic psychedelics (Liechti and Holze, 2022). Despite the many ongoing phase 2 and 3 studies in patients, these aspects of the clinical pharmacology of psilocybin and especially of other psychedelics still require further research (Holze et al., 2023a; van der Graaf, 2023). Acute psychedelic effects can be determined in phase 1 studies and to some extent can be thought of as markers of efficacy because the positive subjective acute effects of psychedelics appear to correlate with the desired therapeutic outcome in patients (Holze et al., 2023b; Roseman et al., 2018; Ross et al., 2016) and lasting improvements in wellbeing in healthy individuals (McCulloch et al., 2022; Schmid and Liechti, 2018). This relationship provides unique opportunities in drug development for dose-finding and optimisation. It allows for a better definition of the dose in phase 1, which will greatly benefit subsequent phase 2 studies and onward. Further dose-finding studies are also needed for each substance and indication in phase 2. Interindividual differences in PK and PD are an issue as with any medication, but moderators of PK and PD can also be tested in phase 1. For example, CYP2D6 is highly varied across the population (Zhou et al., 2017) and has been shown to contribute to the PK of LSD (Vizeli et al., 2021). Conversely, in some studies (Holze et al., 2019; Spriggs et al., 2023) body-weight and BMI do not correlate with the psychedelic effects of a peroral dose of psilocybin or LSD but it is unclear if the therapeutic effects are more tightly associated with the subjective effects than with plasma concentrations or cerebral 5-HT2AR occupancy. No sex differences in the PK, acute subjective, or adverse effects were seen in studies of healthy participants given either psilocybin or LSD (Holze et al., 2019, 2023a) however, an LSD microdosing study reported that women reported experiencing subjective drug effects earlier than men (Bershad et al., 2019).

The cerebral 5-HT2AR density is strongly genetically determined (Pinborg et al., 2008) and decreases significantly with age (Karrer et al., 2019) which may impact drug effects. It has, e.g., been shown that individuals with lower 5-HT2AR binding have longer psychedelic peak and downslope experiences (Stenbæk et al., 2020). Data on age and sex differences, pharmacogenetics, and other moderating factors will be needed from patient studies to determine whether treatment plans should be adjusted.

### 2.2 Comparative pharmacology

Given the various 5-HT2A agonist compounds currently tested in clinical trials and that many more are expected to be tested in the near future, it is critical to determine how these compounds can be compared. Comparisons across different doses of a specific psychedelic or comparisons of different classical psychedelics can be investigated in phase 1 trials (Holze et al., 2022). For example, 20 mg of psilocybin and 0.1 mg of LSD (base) produce roughly equivalent and qualitatively similar self-reported acute effects and may therefore be considered comparable doses (Holze et al., 2022) as do 20 mg of 2C–B and 15 mg of psilocybin (Mallaroni et al., 2023). Such findings are crucial for the interpretation of clinical trials using different psychedelics. For example, the psilocybin dose of 40 mg used in patients with alcohol use disorder (Bogenschutz et al., 2022) can be considered equivalent to the dose of 0.2 mg of LSD used in patients with anxiety disorder (Holze et al., 2023b). Although widely believed that these drugs produce distinct subjective effects even at similar 5-HT2AR occupancy levels, work to date has shown no replicable differences beyond duration. So measures of 5-HT2AR occupancy with positron emission tomography (PET) are required, as well as more precise physiological and psychological measures, e.g., self-reported and cognitive-task based to determine if these differences are veridical (Holze et al., 2022; Mallaroni et al., 2023; Ley et al., 2023). Several drug comparison studies remain missing, as well as comparisons between various routes of administration.

To allow for comparison between laboratories and replication, it would also help to have precise descriptions of the drug formulations used and measurements of plasma drug concentration. If plasma concentrations are not available, the use of similar rating scales across studies to assess the acute psychoactive effects can serve as a proxy to compare the doses used. Such scales include, e.g., simple Visual Analogue Scale/Likert effect measures (“any drug effect”, “good drug effect” and “bad drug effect” repeatedly during acute drug effects). Often used retrospective questionnaires include the 11-dimensional altered states of consciousness questionnaire (11D-ASC) (Studerus et al., 2010), MEQ30 (PES100) (Barrett et al., 2015), and the Emotional Breakthrough Inventory (EBI) (Roseman et al., 2019) which can serve as a psychometric set. The Persisting Effects Questionnaire (Griffiths et al., 2011) and a five-factor personality questionnaire can help characterise persisting effects beyond clinical outcomes. In addition, it has been hypothesised that behavioural changes following psychedelics may contribute to the persisting wellbeing effects in healthy and patient populations (Teixeira et al., 2022) but empirical data characterising lasting effects on behaviour remains an outstanding knowledge gap.

## 3 Phase 2 clinical trials

Most clinical trials investigate whether active treatment is associated with a statistically significant improvement in the symptom scores on scales (e.g., Quick Inventory of Depressive Symptomatology–Self-Report, Montgomery–Åsberg Depression Rating Scale) but this approach has several limitations (Mangalam and Kelty-Stephen, 2022), some of which may be addressable using Baysian methods (Szigeti et al., 2023). Individual data included in some psychedelic research papers shows a wide range of clinical responses to psychedelic treatment (Carhart-Harris et al., 2018). It has been shown that some personality traits (e.g., neuroticism) will negatively influence the treatment outcome (Roseman et al., 2018), while others (e.g., surrendering) positively influence it (Haijen et al., 2018), although these are each preliminary findings that have not been independently replicated. Precision psychiatry is an ambitious but realistic goal that should be applied to psychedelic science (Fernandes et al., 2017). In order to achieve this, data from previously performed and future clinical trials can be pooled and re-examined to understand which subgroups and trait characteristics are predictive of greater treatment response (Moujaes et al., 2023).

### 3.1 Duration and nature of subjective effects

To the extent that the psychedelic experience is needed to achieve therapeutic effects (see also discussion in part 1), there is also a knowledge gap regarding how long the psychedelic experience needs to last to produce lasting therapeutic effects. When administered IV, DMT has a short elimination half-life of 5–6 min (Vogt et al., 2023). These short-acting psychedelic administrations contrast with orally bioavailable psychedelics which have shown clinical efficacy (e.g., psilocybin 5–7 h and LSD 8–16 h (Holze et al., 2022; Gasser et al., 2015; Goodwin et al., 2022)). However the duration of action of DMT can be prolonged by using a slow infusion as was done by Small Pharma Inc who in a recent press release showed encouraging antidepressant effects of I.V. DMT administration in moderate-to-severe major depressive disorder also showed encouraging antidepressant effects at two- and twelve-week follow-up (Cowling, 2023).

In this context, novel data was presented at the meeting. Professor Ramaekers shared the outcome from a small clinical trial investigating the efficacy of the short-acting 5-methoxy-N,N-dimethyltryptamine (5-MeO-DMT) for treatment-resistant depression. Doses between 6 and 36 mg (inhaled) were needed for subjects to report similar subjective psychedelic effects, which lasted 0.25–0.5 h. In this trial, doses were escalated until patients reached a threshold of subjective effects. Seven of eight subjects met remission criteria one week after dosing (Reckweg et al., 2023).

Correlations have been reported between the peak intensity of self-reported subjective effects (using retrospective rating scales such as the MEQ and 5D-ASC) and clinical efficacy (Roseman et al., 2018; Griffiths et al., 2016). Similarly, the “total” subjective effects (i.e., area under the intensity-time curve) could also be associated with clinical efficacy. However, if replicated, these promising efficacy results from short-acting psychedelics could undermine this hypothesis. Further work comparing fast and slow-acting psychedelic formulations will be instrumental to inform our understanding of the peak vs total subjective effects debate. Such work may employ different drugs, or different routes of administration e.g., 2 mg of I.V. psilocybin produces intense subjective effects that last 10–15 min (Carhart-Harris et al., 2012) whereas 25 mg of orally administered psilocybin produces similar subjective effects at peak, but the total experience is stretched over 5–7 h (Holze et al., 2022). If short-acting formulations show similar lasting efficacy compared to longer-lasting formulations, the implications for clinical implementation will be substantial, though ultimately it may be shown that longer experiences are more efficacious or safer for a subset of patients.

Complementary to discussions pertaining to the duration of subjective drug effects is the question of the subjective quality of those effects, and whether these are related to therapeutic effects, either causally or as an epi-phenomenological representation of an underlying therapeutic mechanism. Previous work has shown a link between certain subcomponents of the subjective effects (mystical-type and “oceanic boundlessness”) and symptom reductions (Roseman et al., 2018; Ross et al., 2016; Griffiths et al., 2016; Garcia-Romeu et al., 2015; Davis et al., 2020), whereas other subcomponents of the subjective experience (e.g., anxiety, dissociation) show weaker associations with symptom reduction, or perceived lasting benefits in healthy individuals (ibid.) (McCulloch et al., 2022). Further work delineating *which* subjective effects are related to subsequent symptom reduction, possibly employing qualitative and natural language processing methodologies in addition to the validated psychometric scales alluded to earlier, could lay the groundwork for future therapeutics. These may be either combination therapies (e.g., combination with an anxiolytic to minimise within-session anxiety, or with an empathogen to emphasise positive-mood elements) or structural modifications of known psychedelics. As discussed above, most classical psychedelics appear to have similar subjective effects. However, it was discussed at the meeting that the subjective effects of 5-MeO-DMT may be distinct, although no comparison studies have been performed yet, a clear knowledge gap. Further comparative pharmacology in patient groups, evaluating subjective drug effects and their relation to symptom reductions across a range of psychedelic compounds may elucidate the structure-qualia relationship of psychedelics, enabling next-generation psychedelics to have refined subjective effects emphasising those which produce greater symptom reductions or minimise adverse effects.

### 3.2 Fluid biomarkers

As for many treatments for psychiatric disorders, an important question is if treatment efficacy of psychedelics can be predicted using biological biomarkers. In order to address this, future studies might consider including the evaluation of a standard battery of biomarkers that are known to be modulated in certain psychopathologies are related treatment responses (e.g., neuroplasticity (BDNF) (de Almeida et al., 2019), inflammation (IL-6), and cortisol levels (Sousa et al., 2022)), in easily accessible biological fluids like serum, plasma or saliva. These could then be assessed repeatedly during the day before the first psychedelic session and at the conclusion of the session/s to investigate if there is a link between those markers and therapeutic outcome. Genotyping (e.g., CYP2D6 phenotype) and gene expression analyses focusing, for example, on the 5-HT2AR might be included to gain insight into whether certain genotypes or the change in gene expression patterns may be associated with a greater likelihood of experiencing enduring positive effects (McClure-Begley and Roth, 2022). There is so far, however, no evidence to support that genotypic differences in the 5-HT2AR genes are associated with cerebral 5-HT2AR density (Spies et al., 2020), but epigenetic modifications could play a role in the sustained effects (Knudsen, 2023).

### 3.3 Expectation effects

The extent to which patients' expectations can influence the perceived treatment efficacy is a key knowledge gap. To answer this, it is required that clinical trials try to assess patients’ expectations and prior experience with psychedelic drugs (Aday et al., 2021). Interestingly, a secondary analysis of a recent trial of escitalopram vs psilocybin (Carhart-Harris et al., 2021), showed that expectancy of antidepressant effect was significantly associated with treatment outcome for escitalopram but not psilocybin (unpublished, Erritzoe).

Expectation in RCTs will depend on the frame within which patients give informed consent and the preparation and support offered before, during and after treatment. The ideal is equipoise - equal expectations of the different possible treatments. This is challenging given the hype around the purported benefits of psychedelic experience and that most clinical trials recruit patients by announcing publicly for them, but it is worth remembering that in RCTs, the majority of patients do not respond. Informed consent can honestly downplay the expected benefits of treatment and maintain that attitude through the subsequent psychological support. It is easier to achieve equipoise when the study design includes several active doses and the actual experience at any given dose is so variable. Placebo introduces a different frame and will always invite scepticism except in regard to providing a simple safety baseline.

Related to expectations, some trials have largely excluded patients with previous psychedelic experience. However, where trials do include people with varying levels of psychedelic experience, analysis of subgroups could determine whether people with prior experience have different expectations when entering the study (Aday et al., 2021; Muthukumaraswamy et al., 2021). Similarly, ‘psychotherapy experience’ and an assessment of the patient's own psychological ‘toolkit’, including health behaviours (e.g., sleeping habits), lifestyle (e.g., work-related stressors), and social network, may influence the patient's trajectory during the post-psychedelic ‘integration’ period and should be evaluated to the extent possible.

A key area of discussion in psychedelic medicalisation is the problem of unblinding (Muthukumaraswamy et al., 2021). Future studies may wish to employ study designs to minimise unblinding, utilising specific patient cohorts (e.g., psychedelic naïve) and control groups including psychoactive drugs with minimal therapeutic effects (e.g., d-amphetamine). Those wishing to learn more about psychedelic unblinding may wish to measure unblinding during both peak drug effects and at follow-up, including the reason for their perceived unblinding (e.g., sensory alterations, symptom reductions). During the meeting, representatives from regulatory authorities expressed their concern that unblinding in psychedelic clinical trials can be problematic and that they are open to alternative study designs to provide evidence for the causal therapeutic efficacy of psychedelics as required for regulatory approval. The regulatory authorities encouraged developers to engage with them to discuss such alternative study designs in advance of initiating the trial.

### 3.4 Polypharmacology

Another key question in the clinical implementation of psychedelics is whether the administration of psychedelics is safe and efficacious while patients continue to take their standard antidepressant medication, or if medication should be tapered prior to giving a psychedelic drug. It is clear that antidepressants with significant 5-HT2AR affinity such as amitriptyline, mirtazapine, and trazodone would directly block the agonist activity of serotonergic psychedelics, consistent with the blocking effect that 5-HT2AR antagonists have shown on the subjective effects of psychedelics (Becker et al., 2022; Preller et al., 2017). The same applies to many serotonin receptor blocking antipsychotic drugs that are used to augment SSRI therapy: The most commonly used of these are quetiapine, risperidone, and olanzapine (Zohar et al., 2015). Such drugs may need to be discontinued before psychedelic therapy can be initiated, but as long as it is unknown if the psychedelic experience is a prerequisite for the therapeutic effect, we cannot know if discontinuation is desirable.

Although SSRIs do not directly affect the 5-HT2AR, preclinical studies suggest that SSRI intervention reduces the 5-HT2AR density and function (Celada et al., 2004). Indeed this downregulation has been suggested to be one way in which they might treat depression and anxiety because, in PET studies of humans, higher 5-HT2AR binding has been reported in individuals both at risk for (Frokjaer et al., 2010) and with overt depression (Hrdina et al., 1993; Meyer et al., 2003) although this has not been a consistent finding (Stockmeier, 2003). Data from non-clinical settings tend to show that psychedelic effects are blunted in people who are currently on SSRIs (Gukasyan et al., 2023). However, in a study where healthy volunteers received a two-week intervention with the SSRI escitalopram before dosing with psilocybin (Becker et al., 2022) they did not find that the effect of the psychedelic was blunted, except for small decreases in anxiety. This may be of clinical relevance as SSRI preadministration may facilitate the acceptability of psychedelics in anxious populations. In support of this, the same group has conducted a trial administering a high (200 μg) dose of 10.13039/501100011357LSD to patients in treatment for generalised anxiety disorder on top of their current medicines with significant clinical benefit (Holze et al., 2023b). However, two weeks of SSRI pretreatment may be too short to produce neuro-adaptive changes such as reductions in 5-HT2AR binding or increased synaptic density (Johansen et al., 2023).

Effects found in healthy volunteers do not mean that the same would necessarily be found in depressed people. Because of this uncertainty most clinical trials have withdrawn SSRIs in patients before the psychedelic dosing (Carhart-Harris et al., 2018; Davis et al., 2020). However, this is not without risks – especially worsening of depression and anxiety – that in treatment-resistant patient groups could be worrying (Warner et al., 2006). And for drugs with long half-lives (particularly fluoxetine) tapering can take many weeks.

In a recent trial of psilocybin vs escitalopram (Carhart-Harris et al., 2021) it was shown that patients who had been withdrawn from SSRIs had slightly smaller reductions in depression symptoms following psilocybin than those without prior treatment, even though they had a similar psychedelic experience. However, this is possibly confounded by the re-introduction of escitalopram to one arm and requires replication in larger samples. One hypothesis for this difference is that SSRI treatment immediately prior may lead to neuronal adaptive changes that impede the psychedelic plasticity effects. This question could most easily be investigated in preclinical models. Slower SSRI tapering, e.g. hyperbolic (Horowitz and Taylor, 2019), or partial tapering (to a low dose) should also be tested. There may hypothetically be a sweet spot between efficacy, safety, and convenience offered by a partial reduction in SSRI dose.

It was also explained at the meeting that a longer SSRI pretreatment trial is underway evaluating paroxetine and LSD (NCT05175430) and after the meeting, COMPASS Pathways published a small-scale study showing that the antidepressant effect of psilocybin was not consistently hampered by concomitant use of SSRIs (Goodwin et al., 2023a), but larger studies are warranted.

There is also anecdotal evidence that lithium treatment might increase the adverse effects of psychedelics with a particular risk of seizures (Nayak et al., 2021). Monoamine Oxidase Inhibitors (MAOIs) are generally on a precautionary basis even though they are a component of ayahuasca (a botanical brew containing MAOIs such as harmine and the psychedelic N,N-DMT) which has been shown to be safe (Sousa et al., 2022). As affective disorders are often comorbid with a range of other psychiatric and somatic diseases (Moussavi et al., 2007), drug-drug interaction studies with a wide range of drugs will be essential for widespread safety and efficacy.

### 3.5 Set and setting

In most, if not all, clinical trials with psychedelics an interdisciplinary treatment model has been used, where psychedelic drugs are administered within an integrated framing of psychological support or psychotherapy (Cavarra et al., 2022; Horton et al., 2021). This approach opens an exciting novel venue for integrated treatments but at the same time poses several practical, methodological, and ethical issues that need to be resolved before this treatment model is reflected in future practice. Current psychological models used in clinical trials with psychedelics are multifaceted and layered, typically with a basic support model of preparation, administration, and integration, also sometimes referred to as ‘set’ and ‘setting’ (Johnson et al., 2008), in which a person-centred interpersonal stance is central (Goodwin et al., 2022; Tai et al., 2021). Integrated with this basic support model, different approaches to psychotherapy are also used in clinical trials with psychedelics, e.g., Cognitive Behaviour Therapy (CBT) (Johnson et al., 2017), Motivational Enhancement Therapy (MET) (Bogenschutz et al., 2022; Bogenschutz and Forcehimes, 2017), and Acceptance and Commitment Therapy (10.13039/501100002927ACT) (Sloshower et al., 2020, 2023) or modified ACT-informed support (ACE) (Watts and Luoma, 2020). In some cases, these have been applied in reference to the diagnosis in question, i.e., using MET for alcohol use disorder (Bogenschutz et al., 2022), and in other cases, they have been applied within a trans-diagnostic approach which encompasses a multitude of integrated psychotherapeutic methods, e.g., MDMA-assisted psychotherapy (Mitchell et al., 2023). In the latter, interventions are targeted at the processes of change most relevant to the individual needs of the patient (adhering to the person-centred approach) – in contrast to the diagnostic approach where interventions are focused on the symptoms of a specific diagnostic category (a diagnosis-centred approach). Not all researchers need to take the same position in relation to these issues, but all should be required to state their position and report correspondingly and consistently the psychological setting to allow reproducibility of their studies.

From a purely practical position, the less psychological support is about efficacy and the more it is about safety, the simpler is the proof of a drug effect from a theoretical and regulatory perspective. As will be amplified in the section ‘Regulatory Perspective’ below, medicines are regulated for their efficacy and safety. Where a drug is really designed to ‘assist psychotherapy’ as in the case of MDMA, the more difficult to assess it becomes. For this reason alone, if we are to see re-medicalisation of 10.13039/501100011357LSD or psilocybin, simple psychological support, as appears to be the usual approach in MDD or TRD studies (Goodwin et al., 2023b), is desirable. How the psychedelic opportunity can be augmented by additional targeted psychotherapy will be a challenge for future implementation in actual clinical services.

From a psychological perspective, one of the main issues to be resolved regarding applied psychological models is distinguishing and testing a basic psychological support model against a psychotherapy model. Engaging with one or the other has several implications :1: Ethically, in terms of participants’ consent to the process; when applying a psychotherapy model patients should not only consent to receiving a drug, but also to the type of psychotherapy applied as would be the case in regular psychotherapy, 2: Practically, in terms of focus for the psychological intervention; when using a basic support model focus is on safety related to the drug (drug-centred in addition to non-directive person-centred), whereas in psychotherapy the focus is on the problems presented by the patient in parallel and integrated with drug intervention, and 3: Economically, in terms of costs related to the psychological setting; applying specialised psychotherapy and licensed psychotherapists is likely to be more costly than training health care staff in applying a basic support model.

To date, we have no comparative empirical evidence in favour of either a basic psychological support model or psychotherapy in psychedelic trials. This will be one of the highest priority questions to resolve for future services, and also which specific elements of the applied models are most important, e.g., specific psychotherapeutic approaches, diagnosis-centred, drug-centred or patient-centred, the content of preparation and integration, use of music and type of music during acute administration, number of drug administrations. Another important variable to be examined is the choice of a group versus individual administration model.

We continue to ponder what psychedelic treatment really is and can become: a medical treatment with psychological support?; a psychotherapy assisted by psychedelic medicine?; or an integrated treatment modality? Such ponderations have been the focus of extensive public debate, highlighting this as a key knowledge gap in psychedelic medicalisation (Goodwin et al., 2023b, 2024; Gründer et al., 2023; Bogenschutz, 2024; Schenberg et al., 2024; Alpert et al., 2024; Deckel et al., 2024; Earleywine et al., 2024). Resolving these issues will be central for the potential scaling of psychedelic treatments to the vast number of patients who need effective treatments, in terms of developing scientifically grounded training for assisting personnel and therapists. The psychedelic research field is still young and bringing these issues to mind is therefore vital for the quality of the interdisciplinary research conducted within it. This issue has particular relevance for the regulation and health technology assessment (HTA) of psychedelics. While the regulatory agencies, specifically FDA and EMA, emphasise that they regulate drugs rather than the practice of medicine, the HTA authorities consider the benefits and cost of a specific medical practice.

### 3.6 Regulatory Perspective

#### 3.6.1 European Medicines Agency perspective

The growing interest in the development of psychedelic drugs and their therapeutic potential for the treatment of psychiatric disorders is acknowledged by European regulators and the European Medicines Agency (EMA) and its experts are closely following international research and regulatory activities in this field. However, despite the promising results of recent clinical trials for treatment-resistant depression, PTSD, and substance use disorders and the active promotion of these results by various stakeholders, it was highlighted during the meeting that the requirements for marketing authorisation applications for medicinal products containing active substances classified as psychedelics are the same as for any other marketing authorisation in the EU/EEA. Regulatory approval will require well-designed, randomised, and preferably placebo-controlled studies to demonstrate that potential risks are outweighed by the therapeutic benefits of the product (Butlen-Ducuing et al., 2023).

Due to the safety profile and challenging study setup and execution, it is recommended to start development in a more severely affected population, such as patients with treatment-resistant depression. In this regard, previous failed therapies to define treatment resistance should be defined in the study protocol. Trials to establish short-term efficacy as well as trials to determine the maintenance of effect are needed. The development program will also have to address questions around concomitant psychological support and/or psychotherapy, such as duration of therapy (one session? repeated sessions? – how many and with what interval?), and also the type and modalities of the assisted psychotherapies or psychological support depending possibly also on the type and effect of the psychedelic drug. As in every application, the justification for the adequate therapeutic dose as well as dose-effect relationship are important aspects to address in a marketing authorisation application dossier. In particular, the relationship between characteristics of the acute psychedelic experience and clinical improvement, as well as the need for individualised dosing due to inter-individual variability in drug metabolism, age, sex, or personality should be investigated for the different psychedelics. Real-time measures of drug effects (e.g., using EEG) might also aid this process.

Safety including negative experiences known to be associated with psychedelic states need to be reported thoroughly. Appropriate risk evaluation and mitigation strategies (REMS) supported by data are likely to be required to ensure safe adoption in real practice. The REMS will also include any restrictions on use (e.g., educational material not only for the prescriber but also for those supervising the sessions, as well as consideration around the need for a controlled access program (e.g., as for esketamine previously approved for treatment of treatment-resistant depression in EU)).

The United Nations classification of psychedelic substances as Schedule 1 drugs as adopted in the 1971 Convention on Psychotropic Substances currently prohibits all use, however, it was explained during the meeting that research and therapy with psychedelics are possible according to Article 7 defining special provisions regarding substances in schedule I “**except for scientific and very limited medical purposes** by duly authorised persons, in medical or scientific establishments which are **directly under the control of their Governments** or **specifically approved by them”**. National regulations may have to be adapted, which is possible without violating the convention. Since July 1st, 2023, in Australia MDMA and psilocybin have been rescheduled and so legalised for the treatment of PTSD and treatment-resistant depression, respectively, under highly supervised conditions which includes formal approval by an ethics committee. Other jurisdictions are considering access via a compassionate use program. Rescheduling would facilitate access should these products obtain a marketing authorisation and the evaluation process for reclassification could be initiated at an early stage. Rescheduling is, however, not a purely regulatory decision but in the end dependent on political interest and will.

European regulators are prepared to take a proactive role and offer support for R&D in this field via various scientific and regulatory platforms. Regulatory Guidelines are constantly adapted if scientifically reasonable and the first EMA Guideline to take on board recommendations for psychedelic drug development will be the Guideline on clinical investigations of medicinal products in the treatment of depression. EMA encourages developers to seek early dialogue, stressing that different jurisdictions such as the USA, Europe, Australia, etc. might have divergent views on parts of a clinical development program that need to be dealt with. Engagement with all stakeholders in the field is key to establishing a successful regulatory framework for psychedelic-assisted therapies across the globe.

### 3.7 Health technology assessment perspective

After having obtained a marketing authorisation, the medicinal product will have to undergo a HTA to initiate discussion around pricing and reimbursement. This is particularly challenging in the EU where products can obtain a central marketing authorisation valid in all 27 member states plus Norway and Iceland but will have to undergo HTA, pricing, reimbursement evaluation, and discussions in each of these countries – potentially with more than one payer in each country. Whereas regulatory agencies like EMA are focused on establishing the benefit-risk balance for new therapies HTA/payers are additionally considering the added benefit as compared to existing therapies as well as the impact on overall healthcare budget. Drug developers will have to consider these additional requirements in their development which means designing clinical trials to support relative effectiveness compared to current best treatment options. Moreover, for the discussion around pricing and reimbursement consideration will have to be paid to cost beyond the cost of the medicinal product itself, i.e., diverted cost related to supportive care, hospitalisation, etc. Most HTA bodies offer consultation procedures for developers to address these issues, and there is also an option for a parallel scientific consultation with EU HTA bodies and EMA which could be considered to address expectations from both regulatory bodies and HTA. One paper exploring the cost-effectiveness question for psilocybin versus escitalopram in the UK context has just been published (McCrone et al., 2023).

### 3.8 EMA disclaimer

The views expressed in this document are the personal views of the authors and may not be understood or quoted as being made for or reflecting the position of the EMA or any of its committees or working parties.

## Declaration of competing interest

DEM salary is supported by an unrestricted grant from COMPASS Pathways Ltd. who had no part in the conceptualisation or writing of this manuscript. KPCK is a principal investigator on research projects that are sponsored by Mindmed which is a company developing psychedelic medicines, and she is a paid member of the scientific advisory board of Clerkenwell Health. DJN has advised a number of companies with interests in psychedelic treatments and his research has been supported by the provision of materials by Compass Pathways Usona and Beckley Psytec. GMG is an employee and owns shares and/or options in COMPASS Pathfinder, and has consulted for Beckley Psytech, Boehringer Ingelheim, Clerkenwell Health, EVApharm, H Lundbeck A/S, Janssen Global Services, Novartis, Ocean Neurosciences, Servier, Takeda and WebMD. GG has served as a consultant for Boehringer Ingelheim, Institute for Quality and Efficiency in Health Care (IQWiG), Janssen-Cilag, Lundbeck, Otsuka, Recordati, Roche, ROVI, Sage, and Takeda. He has served on the speakers’ bureau of Gedeon Richter, Janssen Cilag, Lundbeck, Otsuka, Recordati. He has received grant support from 10.13039/100008349Boehringer Ingelheim, 10.13039/501100013327Lundbeck and Saladax. He is co-founder and/or shareholder of Mind and Brain Institute GmbH, Brainfoods GmbH, OVID Health Systems GmbH and MIND Foundation gGmbH. GMK has been a speaker at or consulted for Angelini, Sanos, H. Lundbeck, Onsero, Pangea, Gilgamesh, Pure Technologies, and Abbvie.

## References

[bib1] Aday J., Heifets B.D., Pratscher S.D., Bradley E., Rosen R., Woolley J. (2021). https://psyarxiv.com/nrb3t/.

[bib2] Alpert M.D., O'Donnell K.C., Paleos C.A., Sola E., Stauffer C.S., Wagner A.C. (2024). Psychotherapy in psychedelic treatment: safe, evidence-based, and necessary. Am. J. Psychiatr..

[bib3] Barrett F.S., Johnson M.W., Griffiths R.R. (2015). Validation of the revised Mystical Experience Questionnaire in experimental sessions with psilocybin. J. Psychopharmacol..

[bib4] Becker A.M., Holze F., Grandinetti T., Klaiber A., Toedtli V.E., Kolaczynska K.E. (2022). Acute effects of psilocybin after escitalopram or placebo pretreatment in a randomized, double-blind, placebo-controlled, Crossover study in healthy subjects. Clin. Pharmacol. Ther..

[bib5] Bershad A.K., Schepers S.T., Bremmer M.P., Lee R., de Wit H. (2019). Acute subjective and Behavioral effects of Microdoses of lysergic acid diethylamide in healthy human volunteers. Biol. Psychiatr..

[bib6] Bogenschutz M.P. (2024). Pharmacological and nonpharmacological components of psychedelic treatments: the whole is not the sum of the parts. Am. J. Psychiatr..

[bib7] Bogenschutz M.P., Forcehimes A.A. (2017). Development of a psychotherapeutic model for psilocybin-assisted treatment of alcoholism. J. Humanist. Psychol..

[bib8] Bogenschutz M.P., Ross S., Bhatt S., Baron T., Forcehimes A.A., Laska E. (2022). Percentage of heavy drinking days following psilocybin-assisted psychotherapy vs placebo in the treatment of adult patients with alcohol use disorder. JAMA Psychiatr..

[bib9] Butlen-Ducuing F., McCulloch D.E.W., Haberkamp M., Mattila T., Bałkowiec-Iskra E., Aislaitner G. (2023). The therapeutic potential of psychedelics: the European regulatory perspective. Lancet Lond Engl.

[bib10] Carhart-Harris R.L., Erritzoe D., Williams T., Stone J.M., Reed L.J., Colasanti A. (2012). Neural correlates of the psychedelic state as determined by fMRI studies with psilocybin. Proc. Natl. Acad. Sci. U. S. A..

[bib11] Carhart-Harris R.L., Bolstridge M., Day C.M.J., Rucker J., Watts R., Erritzoe D.E. (2018). Psilocybin with psychological support for treatment-resistant depression: six-month follow-up. Psychopharmacology (Berl).

[bib12] Carhart-Harris R., Giribaldi B., Watts R., Baker-Jones M., Murphy-Beiner A., Murphy R. (2021). Trial of psilocybin versus escitalopram for depression. N. Engl. J. Med..

[bib13] Cavarra M., Falzone A., Ramaekers J.G., Kuypers K.P.C., Mento C. (2022). Psychedelic-assisted psychotherapy—a systematic review of associated psychological interventions. Front Psychol [Internet].

[bib14] Celada P., Puig M.V., Amargós-Bosch M., Adell A., Artigas F. (2004). The therapeutic role of 5-HT1A and 5-HT2A receptors in depression. J. Psychiatry Neurosci..

[bib15] Cowling C. (2023). Small Pharma announces further positive data from SPL026 phase IIa trial in MDD strengthening topline efficacy results [internet]. Small Pharma.

[bib16] Davis A.K., Barrett F.S., May D.G., Cosimano M.P., Sepeda N.D., Johnson M.W. (2020). Effects of psilocybin-assisted therapy on major depressive disorder A randomized clinical trial. JAMA Psychiatr..

[bib17] de Almeida R.N., Galvão AC. de M., da Silva F.S., Silva E.A.D.S., Palhano-Fontes F., Maia-de-Oliveira J.P. (2019). Modulation of serum brain-derived neurotrophic factor by a single dose of ayahuasca: observation from a randomized controlled trial. Front. Psychol..

[bib18] Deckel G.M., Lepow L.A., Guss J. (2024). “Psychedelic assisted therapy” must not Be retired. Am. J. Psychiatr..

[bib19] Earleywine M., De Leo J., Bhayana D., Rajanna B., Scott K. (2024). Psilocybin without psychotherapy: a cart without a horse?. Am. J. Psychiatr..

[bib20] Fernandes B.S., Williams L.M., Steiner J., Leboyer M., Carvalho A.F., Berk M. (2017). The new field of ‘precision psychiatry. BMC Med..

[bib21] Frokjaer V.G., Vinberg M., Erritzoe D., Baaré W., Holst K.K., Mortensen E.L. (2010). Familial risk for mood disorder and the personality risk factor, neuroticism, interact in their association with frontolimbic serotonin 2A receptor binding. Neuropsychopharmacol Off Publ Am Coll Neuropsychopharmacol.

[bib22] Garcia-Romeu A., Griffiths R., Johnson M. (2015). Psilocybin-Occasioned mystical experiences in the treatment of tobacco addiction. Curr. Drug Abuse Rev..

[bib23] Gasser P., Kirchner K., Passie T. (2015). LSD-assisted psychotherapy for anxiety associated with a life-threatening disease: a qualitative study of acute and sustained subjective effects. J. Psychopharmacol..

[bib24] Goodwin G.M., Aaronson S.T., Alvarez O., Arden P.C., Baker A., Bennett J.C. (2022). Single-dose psilocybin for a treatment-resistant episode of major depression. N. Engl. J. Med..

[bib25] Goodwin G.M., Croal M., Feifel D., Kelly J.R., Marwood L., Mistry S. (2023). Psilocybin for treatment resistant depression in patients taking a concomitant SSRI medication. Neuropsychopharmacol Off Publ Am Coll Neuropsychopharmacol.

[bib26] Goodwin G.M., Malievskaia E., Fonzo G.A., Nemeroff C.B. (2023). Must psilocybin always “assist psychotherapy”. Am. J. Psychiatr..

[bib27] Goodwin G.M., Malievskaia E., Fonzo G.A., Nemeroff C.B. (2024). Psychological support for psilocybin treatment: reply to letters on our commentary. Am. J. Psychiatr..

[bib28] Griffiths R.R., Johnson M.W., Richards W.A., Richards B.D., McCann U., Jesse R. (2011). Psilocybin occasioned mystical-type experiences: immediate and persisting dose-related effects. Psychopharmacology (Berl).

[bib29] Griffiths R.R., Johnson M.W., Carducci M.A., Umbricht A., Richards W.A., Richards B.D. (2016). Psilocybin produces substantial and sustained decreases in depression and anxiety in patients with life-threatening cancer: a randomized double-blind trial. J. Psychopharmacol..

[bib30] Gründer G., Brand M., Mertens L.J., Jungaberle H., Kärtner L., Scharf D.J. (2023). Treatment with psychedelics is psychotherapy: beyond reductionism. Lancet Psychiatry [Internet].

[bib31] Gukasyan N., Griffiths R.R., Yaden D.B., Antoine D.G., Nayak S.M. (2023). Attenuation of psilocybin mushroom effects during and after SSRI/SNRI antidepressant use. J Psychopharmacol Oxf Engl.

[bib32] Haijen E.C.H.M., Kaelen M., Roseman L., Timmermann C., Kettner H., Russ S. (2018). Predicting responses to psychedelics: a prospective study. Front Pharmacol [Internet].

[bib33] Holze F., Duthaler U., Vizeli P., Müller F., Borgwardt S., Liechti M.E. (2019). Pharmacokinetics and subjective effects of a novel oral LSD formulation in healthy subjects. Br. J. Clin. Pharmacol..

[bib34] Holze F., Ley L., Müller F., Becker A.M., Straumann I., Vizeli P. (2022). Direct comparison of the acute effects of lysergic acid diethylamide and psilocybin in a double-blind placebo-controlled study in healthy subjects. Neuropsychopharmacology.

[bib35] Holze F., Becker A.M., Kolaczynska K.E., Duthaler U., Liechti M.E. (2023). Pharmacokinetics and pharmacodynamics of oral psilocybin administration in healthy participants. Clin. Pharmacol. Ther..

[bib36] Holze F., Gasser P., Müller F., Dolder P.C., Liechti M.E. (2023). Lysergic acid diethylamide-assisted therapy in patients with anxiety with and without a life-threatening illness: a randomized, double-blind, placebo-controlled phase II study. Biol. Psychiatr..

[bib37] Horowitz M.A., Taylor D. (2019). Tapering of SSRI treatment to mitigate withdrawal symptoms. Lancet Psychiatr..

[bib38] Horton D.M., Morrison B., Schmidt J. (2021). Systematized review of psychotherapeutic components of psilocybin-assisted psychotherapy. Am. J. Psychother..

[bib39] Hrdina P.D., Demeter E., Vu T.B., Sótónyi P., Palkovits M. (1993). 5-HT uptake sites and 5-HT2 receptors in brain of antidepressant-free suicide victims/depressives: increase in 5-HT2 sites in cortex and amygdala. Brain Res..

[bib40] Johansen A., Armand S., Plavén-Sigray P., Nasser A., Ozenne B., Petersen I. (2023). Escitalopram increases synaptic density in the human brain over weeks: a randomized controlled trial. [Internet]. In Review.

[bib41] Johnson M.W., Richards W.A., Griffiths R.R. (2008). Human hallucinogen research: guidelines for safety. J. Psychopharmacol..

[bib42] Johnson M.W., Garcia-Romeu A., Griffiths R.R. (2017). Long-term follow-up of psilocybin-facilitated smoking cessation. Am. J. Drug Alcohol Abuse.

[bib43] Karrer T.M., McLaughlin C.L., Guaglianone C.P., Samanez-Larkin G.R. (2019). Reduced serotonin receptors and transporters in normal aging adults: a meta-analysis of PET and SPECT imaging studies. Neurobiol. Aging.

[bib44] Knudsen G.M. (2023). Sustained effects of single doses of classical psychedelics in humans. Neuropsychopharmacol Off Publ Am Coll Neuropsychopharmacol.

[bib45] Ley L., Holze F., Arikci D., Becker A.M., Straumann I., Klaiber A. (2023). Comparative acute effects of mescaline, lysergic acid diethylamide, and psilocybin in a randomized, double-blind, placebo-controlled cross-over study in healthy participants. Neuropsychopharmacology.

[bib46] Liechti M.E., Holze F. (2022). Dosing psychedelics and MDMA. Curr Top Behav Neurosci.

[bib47] Mallaroni P., Mason N.L., Reckweg J.T., Paci R., Ritscher S., Toennes S.W. (2023). Assessment of the acute effects of 2C-B vs. Psilocybin on subjective experience, mood, and cognition. Clin. Pharmacol. Ther..

[bib48] Mangalam M., Kelty-Stephen D.G. (2022). Ergodic descriptors of non-ergodic stochastic processes. J. R. Soc. Interface.

[bib49] McClure-Begley T.D., Roth B.L. (2022). The promises and perils of psychedelic pharmacology for psychiatry. Nat. Rev. Drug Discov..

[bib50] McCrone P., Fisher H., Knight C., Harding R., Schlag A.K., Nutt D.J. (2023). Cost-effectiveness of psilocybin-assisted therapy for severe depression: exploratory findings from a decision analytic model. Psychol. Med..

[bib51] McCulloch D.E.W., Grzywacz M.Z., Madsen M.K., Jensen P.S., Ozenne B., Armand S. (2022). Psilocybin-induced mystical-type experiences are related to persisting positive effects: a quantitative and qualitative report. Front. Pharmacol..

[bib52] Meyer J.H., McMain S., Kennedy S.H., Korman L., Brown G.M., DaSilva J.N. (2003). Dysfunctional attitudes and 5-HT2 receptors during depression and self-harm. Am. J. Psychiatr..

[bib53] Mitchell J.M., Bogenschutz M., Lilienstein A., Harrison C., Kleiman S., Parker-Guilbert K. (2023). MDMA-assisted therapy for severe PTSD: a randomized, double-blind, placebo-controlled phase 3 study. Focus Am Psychiatr Publ.

[bib54] Moujaes F., Preller K.H., Ji J.L., Murray J.D., Berkovitch L., Vollenweider F.X. (2023). Toward mapping neurobehavioral heterogeneity of psychedelic neurobiology in humans. Biol. Psychiatr..

[bib55] Moussavi S., Chatterji S., Verdes E., Tandon A., Patel V., Ustun B. (2007). Depression, chronic diseases, and decrements in health: results from the World Health Surveys. Lancet Lond Engl.

[bib56] Muthukumaraswamy S.D., Forsyth A., Lumley T. (2021). Blinding and expectancy confounds in psychedelic randomized controlled trials. Expet Rev. Clin. Pharmacol..

[bib57] Nayak S.M., Gukasyan N., Barrett F.S., Erowid E., Erowid F., Griffiths R.R. (2021). Classic psychedelic coadministration with lithium, but not lamotrigine, is associated with seizures: an analysis of online psychedelic experience reports. Pharmacopsychiatry.

[bib58] Pinborg L.H., Arfan H., Haugbol S., Kyvik K.O., Hjelmborg J.V.B., Svarer C. (2008). The 5-HT2A receptor binding pattern in the human brain is strongly genetically determined. Neuroimage.

[bib59] Preller K.H., Herdener M., Pokorny T., Planzer A., Kraehenmann R., Stämpfli P. (2017). The fabric of meaning and subjective effects in LSD-induced states depend on serotonin 2A receptor activation. Curr. Biol..

[bib60] Reckweg J.T., van Leeuwen C.J., Henquet C., van Amelsvoort T., Theunissen E.L., Mason N.L. (2023). A phase 1/2 trial to assess safety and efficacy of a vaporized 5-methoxy-N,N-dimethyltryptamine formulation (GH001) in patients with treatment-resistant depression. Front Psychiatry [Internet].

[bib61] Roseman L., Nutt D.J., Carhart-Harris R.L. (2018). Quality of acute psychedelic experience predicts therapeutic efficacy of psilocybin for treatment-resistant depression. Front Pharmacol [Internet].

[bib62] Roseman L., Haijen E., Idialu-Ikato K., Kaelen M., Watts R., Carhart-Harris R. (2019). Emotional breakthrough and psychedelics: validation of the emotional Breakthrough inventory. J. Psychopharmacol..

[bib63] Ross S., Bossis A., Guss J., Agin-Liebes G., Malone T., Cohen B. (2016). Rapid and sustained symptom reduction following psilocybin treatment for anxiety and depression in patients with life-threatening cancer: a randomized controlled trial. J. Psychopharmacol..

[bib64] Schenberg E.E., King F., da Fonseca J.E., Roseman L. (2024). Is poorly assisted psilocybin treatment an increasing risk?. Am. J. Psychiatr..

[bib65] Schmid Y., Liechti M.E. (2018). Long-lasting subjective effects of LSD in normal subjects. Psychopharmacology (Berl).

[bib66] Sloshower J., Guss J., Krause R., Wallace R.M., Williams M.T., Reed S. (2020). Psilocybin-assisted therapy of major depressive disorder using Acceptance and Commitment Therapy as a therapeutic frame. J Context Behav Sci.

[bib67] Sloshower J., Skosnik P.D., Safi-Aghdam H., Pathania S., Syed S., Pittman B. (2023). Psilocybin-assisted therapy for major depressive disorder: an exploratory placebo-controlled, fixed-order trial. J Psychopharmacol Oxf Engl.

[bib68] Sousa GM de, de Oliveira Tavares V.D., de Menezes Galvão A.C., de Almeida R.N., Palhano-Fontes F., Lobão-Soares B. (2022). Moderators of ayahuasca's biological antidepressant action. Front Psychiatry [Internet].

[bib69] Spies M., Nasser A., Ozenne B., Jensen P.S., Knudsen G.M., Fisher P.M. (2020). Common HTR2A variants and 5‐HTTLPR are not associated with human in vivo serotonin 2A receptor levels. Hum. Brain Mapp..

[bib70] Spriggs M.J., Giribaldi B., Lyons T., Rosas F.E., Kärtner L.S., Buchborn T. (2023). Body mass index (BMI) does not predict responses to psilocybin. J Psychopharmacol Oxf Engl.

[bib71] Stenbæk D.S., Madsen M.K., Ozenne B., Kristiansen S., Burmester D., Erritzoe D. (2020). Brain serotonin 2A receptor binding predicts subjective temporal and mystical effects of psilocybin in healthy humans. J. Psychopharmacol..

[bib72] Stockmeier C.A. (2003). Involvement of serotonin in depression: evidence from postmortem and imaging studies of serotonin receptors and the serotonin transporter. J. Psychiatr. Res..

[bib73] Studerus E., Gamma A., Vollenweider F.X. (2010). Psychometric evaluation of the altered states of consciousness rating scale (OAV). PLoS One.

[bib74] Szigeti B., Phillips L.D., Nutt D. (2023). Bayesian analysis of real-world data as evidence for drug approval: remembering Sir Michael Rawlins. Br. J. Clin. Pharmacol..

[bib84] Szigeti, B., Weiss, B., Rosas, F. E., Erritzoe, D., Nutt, D., & Carhart-Harris, R. (2024). Assessing expectancy and suggestibility in a trial of escitalopram v. psilocybin for depression. *Psychological medicine*, 1–8. Advance online publication. 10.1017/S0033291723003653. 38247730

[bib75] Tai S.J., Nielson E.M., Lennard-Jones M., Johanna Ajantaival R.L., Winzer R., Richards W.A. (2021). Development and evaluation of a therapist training program for psilocybin therapy for treatment-resistant depression in clinical research. Front Psychiatry [Internet].

[bib76] Teixeira P.J., Johnson M.W., Timmermann C., Watts R., Erritzoe D., Douglass H. (2022). Psychedelics and health behaviour change. J Psychopharmacol Oxf Engl.

[bib77] van der Graaf P.H. (2023). Mush room for improving therapeutic approaches in psychiatry. Clin. Pharmacol. Ther..

[bib78] Vizeli P., Straumann I., Holze F., Schmid Y., Dolder P.C., Liechti M.E. (2021). Genetic influence of CYP2D6 on pharmacokinetics and acute subjective effects of LSD in a pooled analysis. Sci. Rep..

[bib79] Vogt S.B., Ley L., Erne L., Straumann I., Becker A.M., Klaiber A. (2023). Acute effects of intravenous DMT in a randomized placebo-controlled study in healthy participants. Transl. Psychiatry.

[bib80] Warner C.H., Bobo W., Warner C., Reid S., Rachal J. (2006). Antidepressant discontinuation syndrome. Am. Fam. Physician.

[bib81] Watts R., Luoma J.B. (2020). The use of the psychological flexibility model to support psychedelic assisted therapy. J Context Behav Sci.

[bib82] Zhou Y., Ingelman‐Sundberg M., Lauschke V. (2017). Worldwide distribution of cytochrome P450 alleles: a meta‐analysis of population‐scale sequencing projects. Clin. Pharmacol. Ther..

[bib83] Zohar J., Stahl S., Moller H.J., Blier P., Kupfer D., Yamawaki S. (2015). A review of the current nomenclature for psychotropic agents and an introduction to the Neuroscience-based Nomenclature. Eur Neuropsychopharmacol J Eur Coll Neuropsychopharmacol.

